# *Bacillus velezensis* BY6 Controls Armillaria Root Rot in Poplar by Reshaping Rhizosphere–Phyllosphere Microbiomes and Inducing Systemic Resistance

**DOI:** 10.3390/microorganisms14030612

**Published:** 2026-03-09

**Authors:** Yasin Shahzaib, Tingliang Zhong, Hongying Yang, Yanxue Xin, Siyu Liu, Kailong Wu, Ping Zhang

**Affiliations:** Laboratory for Exploring and Utilizing Microbial Resources in Cold-Arid Zone Forestry, College of Urban and Environmental Sciences, Shihezi University, Shihezi 832003, China; shahzabe323@stu.shzu.edu.cn (Y.S.); zhongtingliang@stu.shzu.edu.cn (T.Z.); yanghongying@xjshzu.com (H.Y.); xinyanxue@xjshzu.com (Y.X.); 20221012029@stu.shzu.edu.cn (S.L.); 20221012011@stu.shzu.edu.cn (K.W.)

**Keywords:** armillaria root rot, *Bacillus velezensis* BY6, biocontrol agent, poplar root rot, rhizosphere microbiome, phyllosphere microbiome, poplar defense mechanisms, systemic resistance

## Abstract

*Armillaria solidipes,* the causal agent of *Armillaria* root rot, poses a severe and persistent threat to poplar forest plantations. This study evaluated the biocontrol efficacy of the endophytic bacterium *Bacillus velezensis* BY6 against this pathogen and elucidated its multimodal mechanisms of action. BY6 application significantly reduced disease severity by 37.19% at 30 days post-treatment. 16S rRNA (V3–V4) microbiome analysis revealed that BY6 reshaped both the rhizosphere and phyllosphere bacterial communities, consistently enriching beneficial taxa, including *Pantoea ananatis* and members of *Acidobacteria*, while suppressing opportunistic groups. Concurrently, BY6 activated systemic defenses in poplar, evidenced by enhanced activities of key enzymes PAL and POD, and the upregulated expression of SA/JA pathway marker genes (*PR1*, *JAZ*, and *COI1*), coupled with the downregulation of the auxin transporter gene *AUX1*. These data indicate that the biocontrol efficacy of *B. velezensis* BY6 was mediated by a dual mechanism: the modulation of both rhizospheric and phyllospheric bacterial communities, direct elicitation of systemic defense pathways in poplar, which synergistically enhanced resistance against *A. solidipes*.

## 1. Introduction

*Populus davidiana* × *P. alba var. pyramidalis* Louche (Pdpap) is a globally important, fast-growing tree species widely used for timber production and ecological restoration, playing a critical role in carbon sequestration, wood supply, and environmental remediation [1]. However, its productivity is severely threatened by the soil-borne pathogen *Armillaria solidipes*. This disease widely occurs in the main natural poplar forest planting areas in China. The pathogen typically invades host plants via the root system, where it damages cortical and vascular tissues. This damage leads to symptoms such as wilting, leaf chlorosis, biomass reduction, and ultimately plant death, thereby posing a serious challenge to sustainable forest management [2].

Current disease management relies primarily on chemical fungicides such as carbon disulfide-based compounds and methyl bromide [3,4]. Nevertheless, these approaches are not only of limited efficacy, especially against pathogens that have already colonized host root systems, and are associated with soil contamination, the development of pathogen resistance, and significant ecological risks [5]. Consequently, the development of effective, safe, and environmentally friendly alternative strategies has become an urgent priority in contemporary forest disease management.

In recent years, the use of endophytic microorganisms for biological disease control has attracted increasing attention. Species within the *Bacillus* genus have demonstrated considerable potential due to their strong stress tolerance, production of a wide array of antimicrobial compounds, and ability to induce systemic resistance in host plants [6,7]. For instance, *Bacillus velezensis* BY6 was shown to promote the growth of Pdpap and enhance its resistance to *Armillaria solidipes* [8]. Similarly, *Bacillus licheniformis* NB stem 4, isolated from salt-tolerant rice, has been successfully applied to control rice blast caused by *Magnaporthe oryzae* [9]. In addition, a recent study revealed *B. velezensis* Amfr20, isolated from olive roots, exhibited plant growth–promoting traits, tolerance to abiotic stress, and pronounced in vitro antifungal activity [7]. Moreover, the endophytic bacterium *Bacillus amyloliquefaciens* TNOB 22 showed significant inhibitory effects against multiple pathogens responsible for tea plant diseases [10]. Collectively, these studies highlight the broad potential of *Bacillus* endophytes as agents for biological control of plant diseases.

Plants are widely thought to be associated with complex microbial ecosystems in the rhizosphere and phyllosphere, with which they form mutually influential interaction networks. These plant-associated microbiomes facilitate the acquisition and cycling of essential nutrients such as carbon, nitrogen, and phosphorus, enhance plant tolerance to abiotic stresses and pathogens, and thereby support healthy plant growth and development [11]. Endophytic microorganisms play a pivotal role in the root-associated microecosystem, influencing plant growth by modulating nutrient uptake or producing secondary metabolites [12]. Notably, when plants are challenged by pathogenic invasion, they can emit “cry-for-help” signals to the root microbiome, selectively enriching beneficial microorganisms that confer protective effects [13]. This microbial shift can result in a transgenerational enhancement of plant resistance [14,15]. Previous studies have demonstrated that BY6 not only directly inhibits pathogen growth but also promotes Pdpap growth [16]. However, the mechanisms by which BY6 regulates the structure of rhizosphere and phyllosphere microbial communities in Pdpap, and synergistically activates host endogenous defense systems, leading to systemic resistance, remain largely unresolved and warrant further investigation.

Therefore, we hypothesized that BY6 controls Pdpap root rot through a dual mechanism: directly by modulating rhizosphere and phyllosphere microbial communities, and indirectly by activating plant systemic defense responses. To test this hypothesis, we conducted an integrated investigation of bacterial community dynamics, defense enzyme activities, and expression patterns of defense-related genes (*PR1*, *JAZ*, *COI1*, *AUX1*) following BY6 treatment. The novelty of this study lies in its integrative analysis across different plant compartments and biological organization levels; this advances the theoretical framework for its application as a biological control agent and provides a scientific basis for the development of environmentally friendly control strategies against Pdpap root rot.

## 2. Materials and Methods

### 2.1. Source of Strains and Plant Material

The endophytic bacterium *B. velezensis* BY6 used in this study was isolated from the xylem of healthy Pdpap plants and identified by whole-genome sequencing. The genome sequence has been deposited in the NCBI GenBank database under the accession number OP787670.1. The pathogenic fungus *A. solidipes* A2001 was isolated from the roots of diseased Pdpap plants and is currently preserved at the China Forestry Culture Collection Center (CFCC) under the accession number CFCC 70829 [8].

Pdpap seedlings of the cultivar “Shanxin” were obtained from four-week-old sterile tissue-cultured plantlets. The seedlings were transplanted into pots containing loam soil (pH = 7.2) collected from a naturally infested field site and maintained under controlled greenhouse conditions (temperature, 25 ± 2 °C; photoperiod, 16 h light/8 h dark; relative humidity, 60–70%). When the seedlings reached approximately 30 cm in height, they were used for inoculation experiments. A total of 48 Pdpap seedlings were used in this experiment, and two treatment groups were established: (i) a biological control treatment group, in which seedlings were inoculated with BY6 seven days after pathogen inoculation, and (ii) a positive control group, in which seedlings were inoculated with the pathogen alone. Each treatment consisted of eight plants, with three biological replicates.

### 2.2. Disease Index Assessment

From the cultivated seedlings described above, Pdpap seedlings with uniform age (four weeks) and consistent height were selected for pathogenicity assays. The pathogenic fungus *A. solidipes* A2001 and the biocontrol bacterium *B. velezensis* BY6 were first activated on potato dextrose agar (PDA) plates at 26 °C for seven days. Subsequently, mycelial plugs of *A. solidipes* or single colonies of *B. velezensis* BY6 were transferred into potato dextrose (PD) liquid medium and incubated at 26 °C with shaking at 160 rpm for seven days to prepare fermentation cultures. The *B. velezensis* BY6 OD600 concentrations of the resulting suspensions were adjusted to 1.9 × 10^8^–2.0 × 10^8^ CFU mL^−1^ using an Ultraviolet-visible spectrophotometer (Eppendorf D30, Hamburg, Germany), using a hemocytometer (Anxin XB-K-25, Shanghai, China), the concentrations of *A. solidipes* in the resulting suspensions were adjusted to 1.9 × 10^8^–2.0 × 10^8^ spores/mL.

All pots containing Pdpap seedlings were randomly distributed in the greenhouse. The seedlings were first inoculated with *A. solidipes* A2001 by applying 50 mL of fungal fermentation suspension as a soil drench. Seven days later, seedlings in the biological control treatment group were treated with 50 mL of *B. velezensis* BY6 fermentation suspension, whereas seedlings in the positive control group received an equal volume of sterile water. Disease development was monitored regularly after pathogen inoculation, and disease assessments were conducted at 10, 15, and 30 days post-inoculation (which correspond to 3, 8, and 23 days post-BY6 treatment, respectively).

Disease severity was evaluated according to an established root rot grading scale [16]. Based on the extent of root browning and decay as well as aboveground wilting symptoms, disease severity was classified into six levels: Level 0, healthy leaves with no visible symptoms; Level 1, slight browning of leaf tips or margins (<10% of leaf area); Level 2, browning extended to leaf blades (10–30% of leaf area); Level 3, browning or necrosis of the main leaf area (31–50% of leaf area); Level 4, severe leaf blight (51–80% of leaf area) accompanied by leaf chlorosis or wilting; Level 5, complete leaf necrosis and plant death. The disease index (DI) was calculated as follows:

(1) $$Disease\;Index\;\left(\%\right)=[\sum \frac{number\;of\;plants\;at\;each\;severity\;level\;*\;corresponding\;score}{total\;number\;of\;plants\;*\;maximum\;score}\left(100\right)]$$

(2) $$Control\;eficacy\;\left(\%\right)=[\frac{disease\;index\;of\;control\;-\;disease\;index\;of\;treatment}{disease\;index\;of\;control}\left(100\right)]$$

At the same time, the disease assessments (10, 15, and 30 days post-inoculation), rhizosphere soil samples and plant tissue samples were randomly collected from each treatment. Leaf tissues were sampled from the fifth to eighth fully expanded leaves, counted from the apex downward. To minimize contamination by environmental microorganisms, all plant tissue samples were subjected to surface sterilization using the following procedure: samples were first rinsed thoroughly with sterile distilled water for 5 min to remove adhering soil particles, followed by immersion in 75% (*v*/*v*) ethanol for 30 s, rinsing three times with sterile distilled water (5 min each), immersion in 3% (*v*/*v*) sodium hypochlorite solution for 3 min, and a final three rinses with sterile distilled water. Under a laminar flow hood, the surface-sterilized root and leaf tissues were aseptically cut into small segments using sterile scalpels, rapidly frozen in liquid nitrogen, and immediately stored at −80 °C for long-term preservation prior to subsequent analyses.

### 2.3. Enzymatic Activity Assay

Leaf defense enzyme activity was measured at 0, 6, 12, 36, and 72 h post-BY6 treatment. Per replicate, five to eight lower-canopy leaves were pooled, flash-frozen in liquid N_2_, and ground to a fine powder. From this, 0.1 g of tissue was homogenized in 0.9 mL of ice-cold 0.02 M phosphate buffer. The homogenate was vortexed, centrifuged (3000× *g*, 4 min, 4 °C), and the resulting supernatant was used for enzymatic assays, with a 50 µL aliquot per reaction. Catalase (CAT), Peroxidase (POD), and phenylalanine ammonia-lyase (PAL) activity were measured using a commercial kit of Nanjing Jiancheng Bioengineering Institute (Nanjing, China): CAT A007-1-1, POD A084-3, and PAL A137. The absorbance was recorded using a microplate reader (Eppendorf D30, Hamburg, Germany), and the enzyme activity was calculated using the formulae provided by the kits.

### 2.4. Gene Expression Analysis by Quantitative Methods

Total RNA was extracted from poplar leaves at 0, 6, 12, 36, and 72 h post-BY6 treatment to assess transcriptional responses, using the same leaf positions as for the enzyme assays. Following tissue pulverization in liquid nitrogen, RNA was isolated using a Plant RNA Extraction Kit (Bioteke ND5000, Beijing, China). A UV spectrophotometer (Hitach UH4150, Tokyo, Japan) was employed to assess the RNA content and evaluate its purity (OD260/280). A microgram of RNA was reverse transcribed into cDNA using the PrimeScript™ RT Reagent Kit (Takara RR420Q, Tokyo, Japan). Real-time quantitative PCR (RT-qPCR) was performed utilizing SYBR Green Master Mix (Takara RR820A, Tokyo, Japan) in 20 µL reactions comprising 10 µL of master mix, 2 µL of cDNA, 1 µL of primer (10 µM), and 6 µL of ddH_2_O. The Agilent Mx3000P system (Agilent, Santa Clara, CA, USA) was employed for amplification with the following parameters: 95 °C for 10 s, 60 °C for 30 s, and 72 °C for 30 s, repeated 30 cycles. Gene-specific primers for defense-related genes *(COI1*, *JAZ*, *PR1*, *AUX1*) were sourced from Sangon Biotech (Shanghai, China), as previously described [16].

Genomic DNA was extracted from 0.5 g of soil and leaf utilizing a commercial DNA isolation kit. The CTAB/SDS method was employed to isolate genomic DNA, which was subsequently evaluated for quality and concentration by agarose gel electrophoresis and Qubit fluorometry. The V3–V4 (341F/806R) hypervariable region of the 16S rRNA gene was amplified utilizing universal primers that incorporated barcode sequences. Phusion^®^ High-Fidelity PCR Master Mix (New England Biolabs M0531S, Ipswich, MA, USA) was used for PCR, ensuring high amplification efficiency and fidelity. The Qiagen Gel Extraction Kit was used to purify the amplicons. The Truseq^®^ DNA PCR-Free Sample Preparation Kit (Illumina, San Diego, CA, USA) was used to establish libraries. The quality of the libraries was determined using Qubit and qPCR. Qualified libraries were sequenced on an Illumina NovaSeq 2000 platform (Illumina, San Diego, CA, USA), generating paired-end 250 bp (PE250) reads.

### 2.5. Rhizosphere and Phyllosphere Microbial Community Analysis

Raw paired-end reads were demultiplexed, subjected to quality filtering, and then combined utilizing FLASH (v1.2.7) [17]. Chimeric sequences were eliminated via VSEARCH [18]. Utilizing Uparse (v7.0.1001) [18], high-quality sequences (Effective Tags) were categorized into operational taxonomic units (OTUs) at a 97% similarity threshold, and representative sequences were annotated with Mothur against the SILVA 132 database [15]. The alpha diversity indices, including Chao1, ACE, Shannon, Simpson, Good’s coverage, and PD_whole_tree, were calculated using QIIME (v1.9.1) [12]. Statistical comparisons between groups were performed using Wilcoxon and Tukey tests were utilized. Beta diversity analysis was conducted using both weighted and unweighted UniFrac distances, with results visualized using R’s principal coordinate analysis (PCoA), principal component analysis (PCA), and UPGMA clustering functions. Phyllosphere microbial community analysis was conducted in the same manner as the rhizosphere microbiome analysis described above.

### 2.6. Statistical Analysis

All experimental data were subjected to analysis of variance (ANOVA) using the SPSS software v20.0 (IBM Corp., Armonk, NY, USA). The statistical significance of the difference between means was determined using Duncan’s multiple-range test and an independent-sample *t*-test (*p* < 0.05).

## 3. Results

### 3.1. Disease Index Assessment

**Figure 1:**
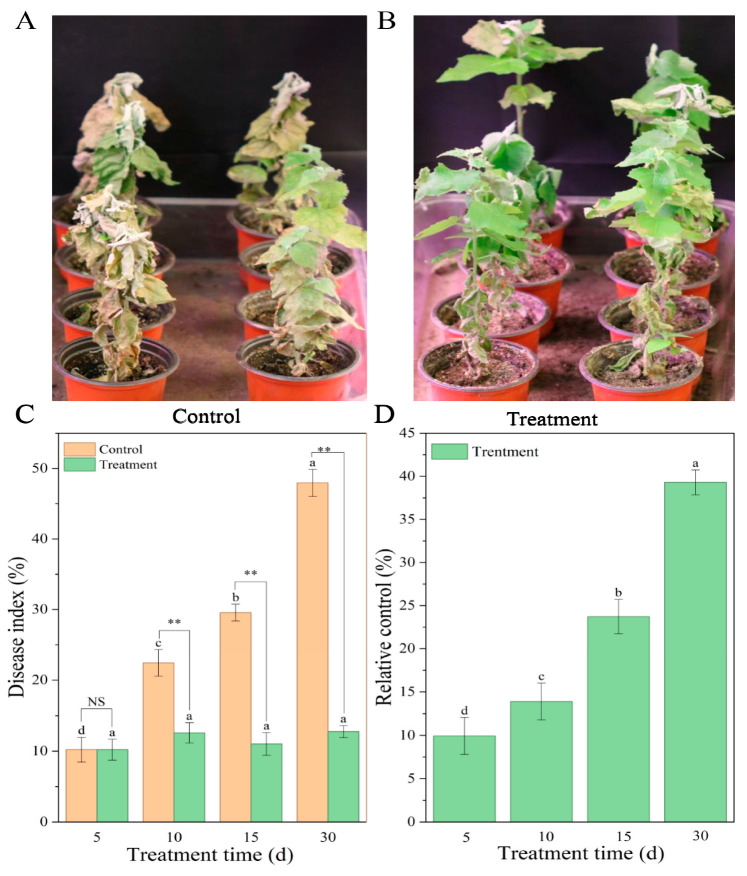
Disease symptoms and disease index of poplar seedlings infected with *A. solidipes*. (**A**) Water-treated control; (**B**) BY6-inoculated treatment. In both (**A**,**B**), seedlings were inoculated with *A. solidipes* 7 days prior to treatment. (**C**) Disease index; (**D**) relative control efficacy. Data represent means ± standard error from four biological replicates. Different lowercase letters (a–d) indicate significant differences among treatments. NS indicates no significant difference (ANOVA, *p* > 0.05); ** indicates a highly significant difference (ANOVA, *p* ≤ 0.01).

At 10 days post-inoculation with *A. solidipes*, disease symptoms observed in “Shanxin” poplar seedlings under controlled conditions were consistent with field observations. In the control group, leaves exhibited chlorosis and wilting, followed by progressive plant death (Figure 1A). By 30 days post-inoculation, the disease index reached 47.96%, which represented an increase of 37.74% compared with day 5 (Figure 1C), These differences were statistically significant (*p* ≤ 0.05, *n* = 4).

In contrast, BY6 application confined wilting to the lower leaves and promoted new growth (Figure 1B). At 30 days post-inoculation, the disease index in the BY6-treated group was reduced by 37.19% compared with the control, and the relative control efficacy exhibited an overall increasing trend, rising by 29.84% from day 5 to reach 39.30% by day 30 (Figure 1D). Overall, these results indicated that BY6 treatment significantly reduced the disease index of *A. solidipes*–infected “Shanxin” poplar seedlings, demonstrating a sustained biocontrol activity.

### 3.2. Enzyme Activities (CAT, PAL, POD)

**Figure 2:**
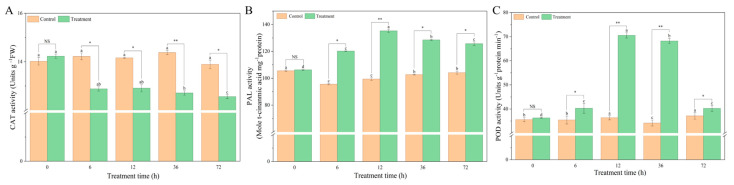
Effects of BY6 on defense enzyme activities in poplar seedlings. (**A**) CAT; (**B**) PAL; (**C**) POD. Data represent means ± standard error from four biological replicates. Different lowercase letters (a–d) indicate significant differences among treatments. NS indicates no significant difference (ANOVA, *p* > 0.05); * indicates a significant difference (ANOVA, *p* ≤ 0.05); ** indicates a highly significant difference (ANOVA, *p* ≤ 0.01).

As shown in Figure 2, in BY6-treated Pdpap seedlings, CAT activity decreased at 0, 6, 12, 36, and 72 h, reaching a minimum at 36 h, which was 0.88-fold lower than at 0 h. In contrast, no significant differences were observed among time points in the control group. PAL activity in the BY6-treated group increased overall at 0, 6, and 12 h, reaching a maximum at 12 h, which was 1.27-fold higher than the activity at 0 h, while no significant changes were detected in the control group. Peroxidase (POD) activity in BY6-treated seedlings exhibited an increase at 6, 12, and 36 h, peaking at 12 h with a 1.94-fold increase relative to 0 h. In the control group, POD activity showed no significant differences at 6, 12, or 36 h, with only slight increases at 12 and 36 h. These results collectively indicate that BY6 treatment initially enhanced POD and PAL activity in Pdpap seedlings, thereby strengthening the seedlings’ resistance to *A. solidipes* infection during the early stages of pathogen challenge.

### 3.3. Gene Expression Patterns (COI1, JAZ, PR1, AUX1)

**Figure 3:**
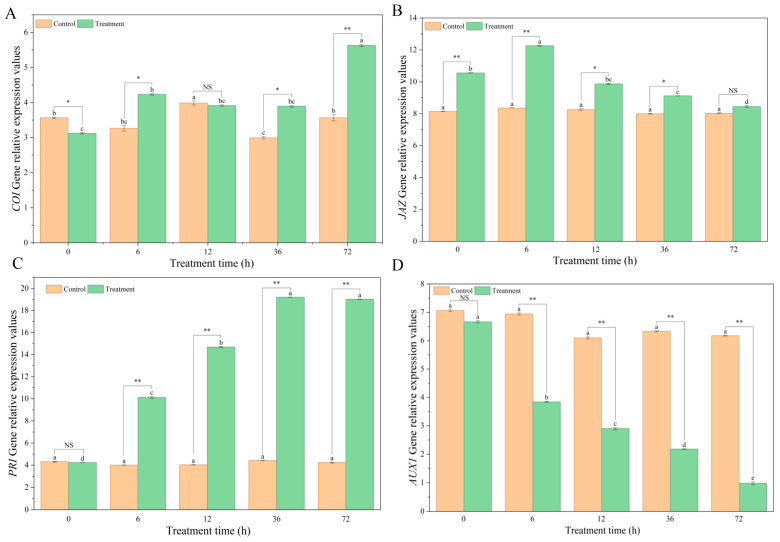
Expression patterns of disease-resistance-related genes in poplar seedlings following BY6 treatment. (**A**) *COI1*; (**B**) *JAZ*; (**C**) *PR1*; (**D**) *AUX1*. Data represent means ± standard error from four biological replicates. Different lowercase letters (a–d) indicate significant differences among treatments (ANOVA, *p* < 0.05). NS indicates no significant difference (ANOVA, *p* > 0.05); * indicates a significant difference (ANOVA, *p* ≤ 0.05); ** indicates a highly significant difference (ANOVA, *p* ≤ 0.01).

The expression of key genes involved in disease resistance signaling pathways in Pdpap seedlings was next analyzed. As shown in Figure 3A, *COI1* expression in the BY6-treated group was upregulated, reaching a maximum transcript level at 72 h, which was 1.58-fold higher than in the control group. BY6 treatment induced distinct temporal expression patterns for the two defense-related genes. *JAZ* expression showed a transient peak at 6 h, while *PR1* exhibited sustained upregulation, culminating in a 4.44-fold higher expression than the control at 36 h (Figure 3B,C). In contrast, no significant changes were observed in the control group for these three genes. As shown in Figure 3D, *AUX1* expression in the BY6-treated group showed a downregulation trend, reaching its lowest transcript level at 72 h, whereas expression in the control group remained largely unchanged, with no significant differences observed.

### 3.4. Rhizosphere Microbial Community Analysis

**Figure 4:**
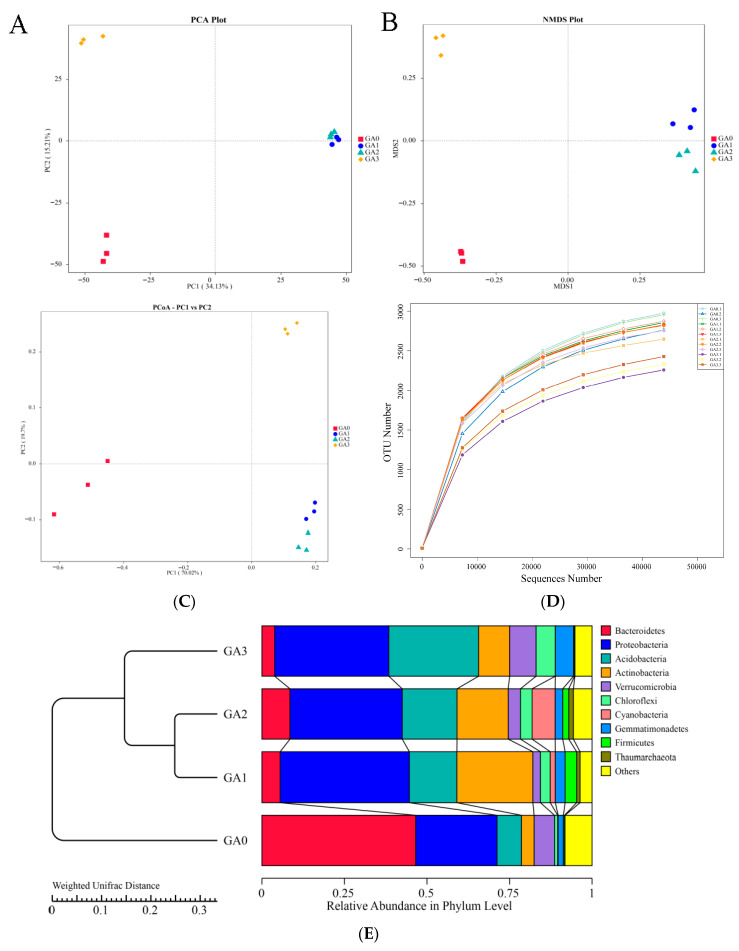
Effects of BY6 on rhizosphere bacterial community structure in diseased Pdpap seedlings at different time points. (**A**) Principal Component Analysis (PCA); (**B**) Non-Metric Multidimensional Scaling (NMDS); (**C**) Principal Coordinates Analysis (PCoA); (**D**) Operational Taxonomic Unit (OTU) richness, with the *x*-axis representing the number of randomly selected sequencing reads per sample and the *y*-axis representing the corresponding number of OTUs. Different samples are represented by curves of different colors; (**E**) Unweighted Pair-Group Method with Arithmetic Mean (UPGMA) clustering. CA0, CA1, CA2, and CA3 represent rhizosphere samples of diseased Pdpap seedlings treated with BY6 at 0, 10, 15, and 30 days, respectively. Analyses were based on 16S rRNA gene sequencing data.

A total of 843,372 raw sequences were obtained from 16S rRNA gene sequencing of rhizosphere bacterial communities in diseased Pdpap seedlings treated with BY6. After quality control and sequence assembly, 806,782 high-quality sequences were retained, and filtering produced 734,725 effective sequences. PCA of the rhizosphere bacterial community (Figure 4A) revealed that samples from the control at 0 days (CA0) clustered distinctly from the 10, 15, and 30-day samples (CA1, CA2, and CA3), with tight clustering of replicates within each group. PC1 and PC2 accounted for 49.34% of the community variation. NMDS analysis (Figure 4B) showed similar patterns, with CA0 clearly separated from CA1, CA2, and CA3, and samples within each group clustered tightly. PCoA based on the Bray–Curtis distance matrix (Figure 4C) further confirmed temporal shifts in the rhizosphere bacterial community following BY6 treatment, with CA0 clearly separated from CA1, CA2, and CA3. In addition, PC1 and PC2 accounted for 89.72% of the variation in rhizosphere bacterial diversity. OTU-based rarefaction curves (Figure 4D) indicated that the curves for all samples approached a plateau, with the number of observed taxa (i.e., distinguishable microbial units) no longer increasing sharply with sequencing depth, suggesting that the sequencing effort was sufficient to capture rhizosphere bacterial diversity. UPGMA clustering of the rhizosphere bacterial communities at different time points (Figure 4E) yielded results consistent with PCoA, indicating that BY6 treatment affected the composition of rhizosphere microbial communities in diseased Pdpap seedlings. Intra-group variation was detectable among replicates within each treatment. Following BY6 application, temporal dynamics in the assembly of rhizosphere bacterial communities were observed.

**Figure 5:**
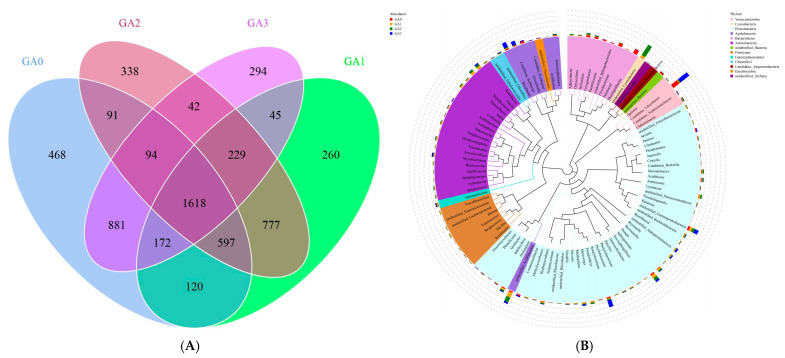
Effects of BY6 on rhizosphere bacterial community OTUs and genus-level phylogeny in diseased Pdpap seedlings at different time points. (**A**) Venn diagram showing OTU overlap among different treatment groups. The number in the center represents OTUs shared among groups, while numbers within each circle indicate unique OTUs specific to that group, excluding shared OTUs. (**B**) Phylogenetic tree constructed using representative sequences at the genus level. Branches and sector colors correspond to different phyla, and the stacked bar charts surrounding the outer ring represent the relative abundance of each genus in different samples.

BY6 influenced the rhizosphere bacterial community structure of diseased Pdpap seedlings, as revealed by OTU-based Venn analysis. As shown in Figure 5A, a total of 6026 OTUs were identified across the four treatment groups, with 1618 OTUs shared among all groups. Unique bacterial OTUs declined from 468 at day 0 (CA0) to 260 at day 30 (CA3), reflecting a reduction in rhizosphere alpha diversity over time. Total OTU richness also shifted temporally, with the order CA0 > CA2 > CA3 > CA1, further indicating BY6-induced community restructuring.

To further investigate phylogenetic relationships at the genus level, representative sequences for the top 100 genera were obtained via multiple sequence alignment and are shown in Figure 5B. In terms of community composition, *Proteobacteria* exhibited the highest relative abundance with 45 genera, followed by *Actinobacteria* with 19 genera, together constituting the dominant phyla across the samples. Other commonly observed rhizosphere microbial taxa included *Cyanobacteria* (1 genus), *Acidobacteria* (7 genera), and *Bacteroidetes* (9 genera). Notably, several clades in the phylogenetic tree were labeled as “*Unidentified*”, indicating the presence of unclassified or poorly characterized taxa within the rhizosphere.

**Figure 6:**
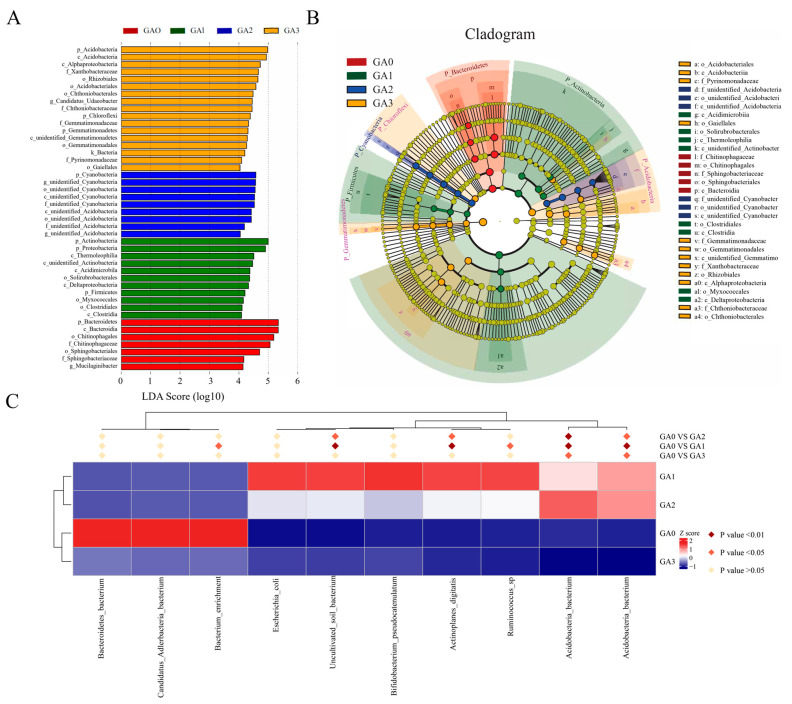
Significant differences in rhizosphere bacterial taxa of diseased Pdpap seedlings at different time points following BY6 treatment. (**A**) Linear Discriminant Analysis (LDA) scores of rhizosphere bacteria (LDA > 3.5). (**B**) Phylogenetic cladogram from LEfSe analysis. The cladogram displays taxonomic abundance from phylum to genus/species (from inner to outer circles), with circle size representing relative abundance and bar width corresponding to the LDA score, indicating the degree of difference among groups. (**C**) Heatmap showing the distribution of differential taxa across samples in different treatment groups.

LEfSe analysis (LDA ≥ 3.0, *p* < 0.05) was performed to identify differentially abundant bacterial taxa among samples (Figure 6A), yielding 44 significant biomarkers. At 0 days (CA0), 7 significant differentially abundant taxa were identified, with the top two being p_Bacteroidetes and c_Bacteroidia. At 10 days (CA1), 11 differential taxa were detected, with p_Actinobacteria and p_Proteobacteria ranking highest. At 15 days (CA2), 9 differential taxa were identified, with p_Cyanobacteria and g_Unidentified_Cyanobacteria being the top two. At 30 days (CA3), 17 significantly different taxa were detected, with p_Acidobacteria and c_Acidobacteriia ranking highest. LEfSe analysis of GA0–GA1–GA2–GA3 (Figure 6B) identified 31 biomarkers with LDA scores > 3.5, including o_Acidobacteriales, c_Acidobacteriia, and f_Pyrinomonadaceae. These LEfSe results were consistent with the findings of community composition and differential abundance analyses, further confirming that BY6 treatment influenced the temporal dynamics and relationships of rhizosphere bacterial communities in diseased Pdpap seedlings.

To further investigate the relationships among differential taxa across groups, a heatmap was generated to visualize their distribution across samples in different treatment groups (Figure 6C). Between 0 days (CA0) and 10 days (CA1), significant differentially abundant taxa included *Acidobacteria_bacterium*, *Ruminococcus_sp*, *Actinoplanes_digitatis*, *Uncultivated_soil_bacterium*, and *Bacterium_enrichment*, whereas non-significant taxa included *Bifidobacterium_pseudocatenulatum*, *Escherichia_coli*, *Candidatus_Adlerbacteria_bacterium*, and *Bacteroidetes_bacterium*. Between 0 days (CA0) and 15 days (CA2), significant differentially abundant taxa were *Acidobacteria_bacterium*, *Acidobacteria_bacterium*, *Actinoplanes_digitatis*, and *Uncultivated_soil_bacterium*, while non-significant taxa included *Ruminococcus_sp*, *Bifidobacterium_pseudocatenulatum*, *Escherichia_coli*, *Bacterium_enrichment*, *Candidatus_Adlerbacteria_bacterium*, and *Bacteroidetes_bacterium*. Between 0 days (CA0) and 30 days (CA3), the significant differentially abundant taxa were *Acidobacteria_bacterium* and *Acidobacteria_bacterium*, whereas non-significant taxa included *Ruminococcus_sp*, *Actinoplanes_digitatis*, *Bifidobacterium_pseudocatenulatum*, *Uncultivated_soil_bacterium*, *Escherichia_coli*, *Bacterium_enrichment*, *Candidatus_Adlerbacteria_bacterium*, and *Bacteroidetes_bacterium*. Across all three comparisons, *Acidobacteria_bacterium* was consistently identified as a dominant taxon.

**Figure 7:**
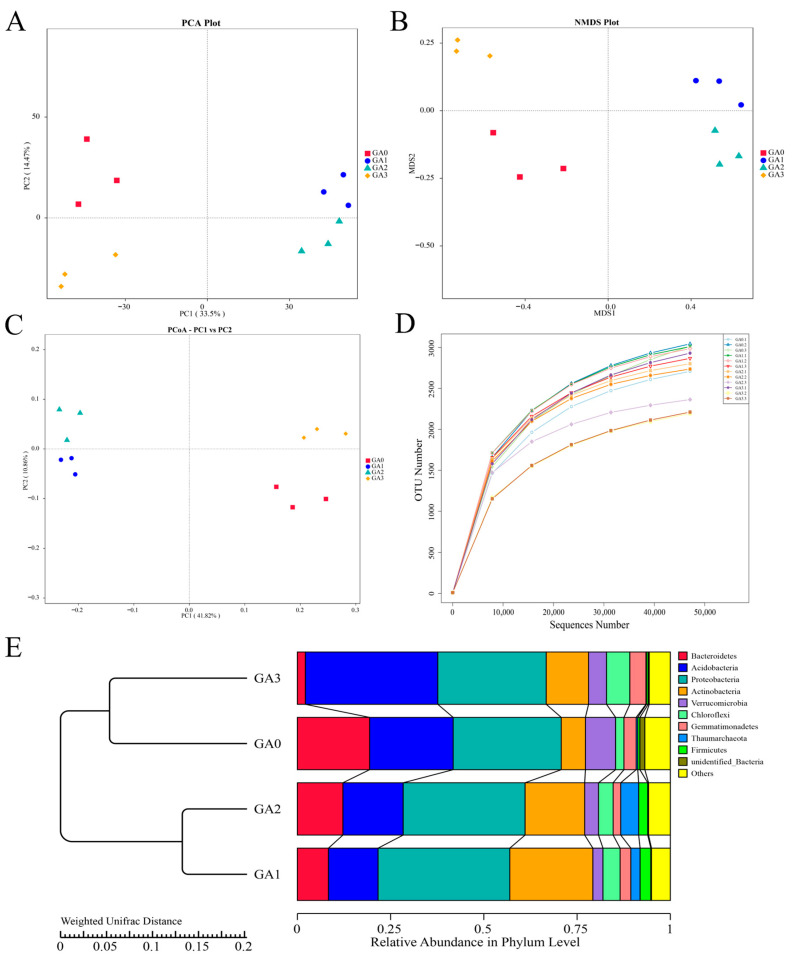
Effects of BY6 on phyllosphere bacterial community structure in diseased Pdpap seedlings at different time points. (**A**) Principal Component Analysis (PCA); (**B**) Non-Metric Multidimensional Scaling (NMDS); (**C**) Principal Coordinates Analysis (PCoA); (**D**) Operational Taxonomic Unit (OTU) richness, with the *x*-axis representing the number of randomly selected sequencing reads per sample and the *y*-axis representing the corresponding number of OTUs. Different samples are indicated by curves of different colors; (**E**) Unweighted Pair-Group Method with Arithmetic Mean (UPGMA) clustering. CA0, CA1, CA2, and CA3 represent phyllosphere samples of diseased Pdpap seedlings treated with BY6 at 0, 10, 15, and 30 days, respectively. Analyses were based on 16S rRNA gene sequencing data.

A total of 887,862 raw sequences were obtained from 16S rRNA gene sequencing of phyllosphere bacterial communities in diseased Pdpap seedlings treated with BY6. After quality control and sequence assembly, 873,798 high-quality sequences were retained, and 776,679 effective sequences were obtained after filtering. PCA of phyllosphere bacteria (Figure 7A) revealed that samples from day 0 (CA0) clustered distinctly from those at later time points (CA1, CA2, CA3), while replicates within each group exhibited high consistency. PC1 and PC2 accounted for 49.97% of the variation in community composition. NMDS analysis (Figure 7B) showed a similar pattern, with CA0 distinctly separated from CA1, CA2, and CA3. Samples within each group clustered closely, although differences within groups were not significant. PCoA based on the Bray–Curtis distance matrix (Figure 7C) further confirmed temporal differences in phyllosphere bacterial community structure following BY6 treatment, with CA0 clearly separated from CA1, CA2, and CA3. PC1 and PC2 accounted for 52.68% of the phyllosphere bacterial diversity. OTU-based rarefaction curves (Figure 7D) indicated that all sample curves approached a plateau, and the number of observed taxa (i.e., distinguishable microbial units) no longer increased sharply with sequencing depth, suggesting that the sequencing depth was sufficient to capture the microbial diversity of the samples. UPGMA clustering of phyllosphere bacterial communities at different time points (Figure 7E) yielded results consistent with PCoA, indicating that BY6 treatment influenced the composition of phyllosphere microbial communities in diseased Pdpap seedlings. Intra-group variation was detectable among replicates within each treatment. BY6 treatment triggered distinct temporal changes in the structure of phyllosphere bacterial communities.

**Figure 8:**
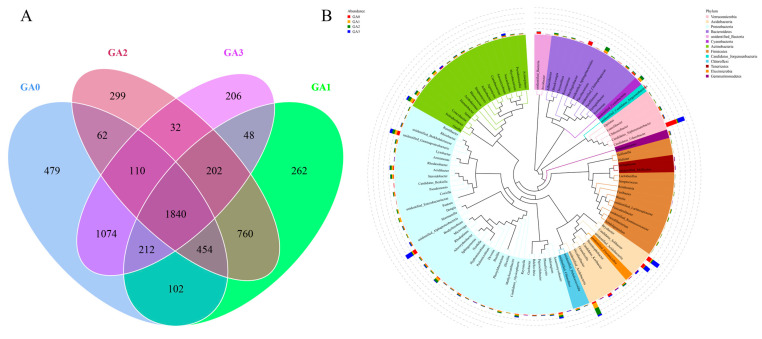
Effects of BY6 on phyllosphere bacterial OTUs and genus-level phylogeny in diseased Pdpap seedlings at different time points. (**A**) Venn diagram showing OTU overlap among different treatment groups. The number in the center represents OTUs shared among groups, while numbers within each circle indicate unique OTUs specific to that group, excluding shared OTUs. (**B**) Phylogenetic tree constructed using representative sequences at the genus level. Branches and sector colors correspond to different phyla, and the stacked bar charts surrounding the outer ring represent the relative abundance of each genus in different samples.

BY6 influenced the phyllosphere bacterial community structure of diseased Pdpap seedlings, as revealed by OTU-based Venn analysis. As shown in Figure 8A, a total of 6142 OTUs were identified across the four treatment groups, with 1840 OTUs shared among all groups. The number of unique OTUs in the phyllosphere decreased from 479 at day 0 (CA0) to 262 at day 30 (CA3), with intermediate values of 299 at day 10 (CA1) and 206 at day 15 (CA2). Compared with CA0, the number of phyllosphere OTUs decreased notably at 10, 15, and 30 days following BY6 treatment. Across the three later time points, the total OTU counts ranked from highest to lowest as CA0 (0 days) > CA2 (15 days) > CA1 (10 days) > CA3 (30 days), indicating temporal shifts in phyllosphere bacterial diversity after BY6 application.

To further investigate phylogenetic relationships at the genus level, representative sequences of the top 100 genera were obtained through multiple sequence alignment and are shown in Figure 8B. In terms of community composition, *Proteobacteria* exhibited the highest relative abundance with 38 genera, followed by *Actinobacteria* with 16 genera, together constituting the dominant phyla in the samples. Other commonly observed phyllosphere microbial taxa included *Cyanobacteria* (11 genera), *Acidobacteria* (8 genera), and *Bacteroidetes* (11 genera). Notably, several clades in the phylogenetic tree were labeled as “Unidentified_”, indicating the presence of unclassified or poorly characterized taxa in the phyllosphere microbiome.

**Figure 9:**
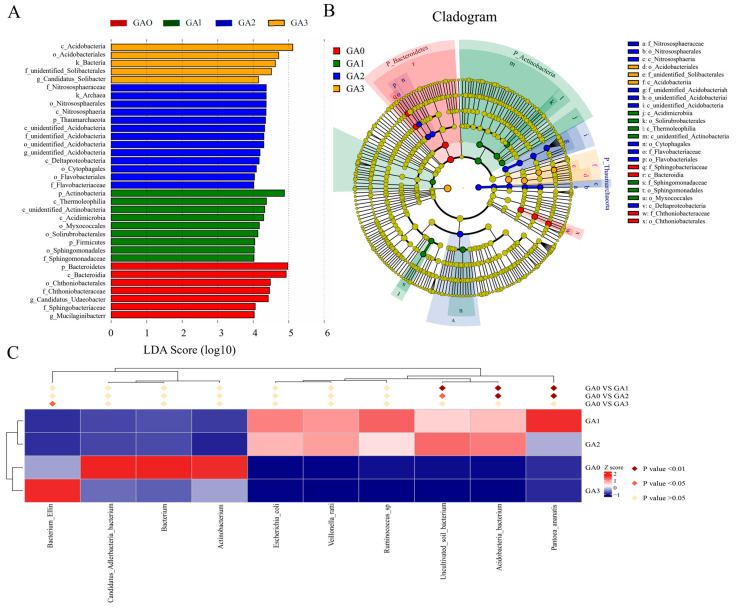
Significant differences in phyllosphere bacterial taxa of diseased Pdpap seedlings at different time points following BY6 treatment. (**A**) Linear Discriminant Analysis (LDA) scores of phyllosphere bacteria (LDA > 3.5). (**B**) Phylogenetic cladogram from LEfSe analysis. The cladogram displays taxonomic abundance from phylum to genus/species (from inner to outer circles), with circle size representing relative abundance and bar width corresponding to the LDA score, indicating the magnitude of differences among groups. (**C**) Heatmap showing the distribution of differential taxa across samples in different treatment groups.

LEfSe analysis (LDA ≥ 3.0, *p* < 0.05) was performed to identify differences in bacterial taxa among phyllosphere samples (Figure 9A). A total of 34 significant differentially expressed taxa were detected across the groups. At 0 days (CA0), 7 significantly different taxa were identified, with the top two being p_Bacteroidetes and c_Bacteroidia. At 10 days (CA1), 9 differential taxa were detected, with p_Actinobacteria and c_Thermoleophilia ranking highest. At 15 days (CA2), 13 differential taxa were identified, with the top three being f_Nitrososphaeraceae, k_Archaea, and o_Nitrososphaerales. At 30 days (CA3), 5 significantly different taxa were detected, with c_Acidobacteriia and o_Acidobacteriales ranking highest. LEfSe analysis across GA0–GA1–GA2–GA3 (Figure 9B) identified 24 biomarkers with LDA scores > 4, including f_Nitrososphaeraceae, o_Nitrososphaerales, and c_Nitrososphaeria. These LEfSe results were consistent with findings from community composition and differential abundance analyses, further confirming that BY6 treatment influenced the temporal dynamics and interactions of phyllosphere bacterial communities in diseased Pdpap seedlings.

To further explore the relationships among differential taxa in phyllosphere microbial communities, a heatmap was generated to visualize their distribution across samples in different treatment groups (Figure 9C). Between 0 days (CA0) and 10 days (CA1), the significantly different taxa were *Pantoea_ananatis*, *Acidobacteria_bacterium*, and *Uncultivated_soil_bacterium*, whereas non-significant taxa included *Ruminococcus_sp*, *Veillonella_ratti*, *Escherichia_coli*, *Actinobacterium*, *Bacterium*, *Candidatus_Adlerbacteria_bacterium*, and *Bacterium_Ellin*. Between 0 days (CA0) and 15 days (CA2), the significantly different taxa were *Pantoea_ananatis*, *Acidobacteria_bacterium*, and *Uncultivated_soil_bacterium,* while non-significant taxa included *Veillonella_ratti*, *Ruminococcus_sp*, *Escherichia_coli*, Actinobacterium, Bacterium, *Candidatus_Adlerbacteria_bacterium*, and *Bacterium_Ellin*. Between 0 days (CA0) and 30 days (CA3), the only significantly different taxon was *Bacterium_Ellin*, with all other taxa showing no significant differences. Across all three comparisons, *Pantoea_ananatis*, *Acidobacteria_bacterium*, and *Uncultivated_soil_bacterium* were consistently identified as dominant taxa.

## 4. Discussion

Armillaria root rot is a severe soil-borne disease that poses a significant threat to Pdpap, and the pathogen’s ability to persist in soil makes its control particularly challenging. The rhizosphere and phyllosphere, as critical interfaces for plant–soil–microbe interactions, harbor microbial communities that are considered the first line of defense against soil-borne pathogens and play a central role in maintaining plant health [19,20,21]. In this study, we systematically analyzed the effects of the endophytic bacterium BY6 on rhizosphere and phyllosphere bacterial community structure, the responses of key defense-related enzymes, and the expression patterns of defense-associated genes (*PR1*, *JAZ*, *COI1*, and *AUX1*). Importantly, we sought to elucidate the biocontrol mechanisms of BY6 and provide a theoretical basis for microbiome-based, environmentally friendly management of forestry diseases.

Unlike previous studies on BY6, this work reveals for the first time the molecular mechanism by which this endophytic bacterium activates systemic resistance in Pdpap, namely through significant upregulation of SA/JA signaling core marker genes, including *PR1*, *JAZ*, and *COI1*. *PR1* is a classical marker of the SA pathway, and its expression is directly associated with the establishment of systemic acquired resistance (SAR). Similarly, in *Arabidopsis*, SA accumulation has been shown to markedly induce *PR1* expression, thereby enhancing resistance to pathogens and tolerance to drought stress [22]. Meanwhile, within the JA signaling pathway, the F-box protein *COI1* serves as the receptor for jasmonoyl-isoleucine (JA-Ile) and a component of the SCF^*COI1* E3 ubiquitin ligase complex. *COI1* mediates the degradation of *JAZ* transcriptional repressors, thereby releasing transcription factors to activate downstream defense genes [23]. Collectively, these molecular events form the signaling basis for BY6-induced resistance in Pdpap.

On the other hand, BY6 treatment suppressed the expression of the auxin transport carrier gene *AUX1*. Downregulation of auxin signaling suggests a shift in resource allocation from growth toward defense, consistent with the classical “growth–defense trade-off” theory [24]. The observed transcriptional changes correlated with effective disease control. BY6 application resulted in a 39.45% reduction in disease index at 30 days post-treatment, contrasting with the progressive disease development in untreated controls At the physiological level, BY6 treatment also significantly enhanced the activities of PAL and POD, while modulating CAT activity. PAL is a rate-limiting enzyme in the phenylpropanoid pathway, responsible for the synthesis of lignin, phytoalexins, and other antimicrobial compounds. POD and CAT are key enzymes in the reactive oxygen species (ROS) scavenging system. Their synergistic activity helps Pdpap maintain ROS homeostasis during the early stages of pathogen infection, allowing ROS bursts to mediate defense while minimizing oxidative damage [25]. In this study, the coordinated increase in POD and CAT activities suggests that BY6 may enhance Pdpap’s ability to regulate ROS metabolism and mitigate oxidative stress.

In this study, microbial diversity decreased from the rhizosphere to the phyllosphere, yet both rhizosphere and phyllosphere bacterial communities exhibited dynamic responses to Armillaria infection. PCoA based on Bray–Curtis distances revealed that, in both healthy and diseased Pdpap, rhizosphere and phyllosphere bacterial communities were separated along the first principal component, indicating that the BY6 treatment significantly altered the microbial community structure of infected seedlings. At the phylum level, both rhizosphere and phyllosphere communities were dominated by *Actinobacteria*, *Proteobacteria, Acidobacteria*, and *Firmicutes*, although the relative abundances of certain dominant taxa differed significantly between healthy and diseased plants. Specifically, after induction by the endophyte BY6, the relative abundances of *Proteobacteria*, *Acidobacteria*, *Actinobacteria*, *Verrucomicrobia*, and *Bacillota* in the rhizosphere soil of diseased Pdpap were higher than in the control (GA0). Previous studies have shown that enrichment of *Proteobacteria* in the tomato rhizosphere can reduce the incidence of bacterial wilt, whereas pathogen infection perturbs this phylum [26]. Meanwhile, *Actinobacteria*, a common biocontrol resource against soil-borne pathogens, play a key role in plant disease resistance through their rhizosphere composition [27]. In this study, both *Proteobacteria* and *Actinobacteria* in the BY6-induced Pdpap rhizosphere exhibited high relative abundances that increased significantly over time, a phenomenon also reported in potato infected with *Rhizoctonia solani* [28]. Similarly, BY6 induction altered the phyllosphere bacterial composition: the relative abundances of *Actinobacteria*, *Verrucomicrobia*, and *Firmicutes* in the leaves of diseased seedlings were significantly higher than in the control, whereas *Proteobacteria* and *Acidobacteria* were significantly reduced. Overall, these patterns reveal that BY6 treatment triggers both shared and tissue-specific microbial responses between the rhizosphere and phyllosphere.

This study demonstrated that inoculation with the endophytic bacterium BY6 significantly reshaped the bacterial community structure in both the rhizosphere and phyllosphere of Pdpap. The widespread increase in the relative abundances of beneficial bacterial taxa, such as Actinobacteria, Verrucomicrobia, and Firmicutes, suggests that BY6 may directly or indirectly optimize the plant’s microbial symbiosis network, thereby enhancing systemic disease resistance. Numerous studies have shown that Bacillus species can not only directly suppress pathogenic microorganisms but also activate and recruit beneficial microbes to the rhizosphere and phyllosphere by inducing plant immune responses and secreting specific metabolites, forming a synergistic defense network [29,30].

At the genus level, *Pantoea ananatis* emerged as a shared dominant taxon in both the rhizosphere and the phyllosphere following BY6 treatment, which is particularly significant. This bacterium is a well-recognized biocontrol agent that produces indole and other antimicrobial compounds to directly inhibit pathogens. Its genome also contains multiple gene clusters for the biosynthesis of siderophores, antimicrobial peptides, and other secondary metabolites, conferring broad-spectrum disease suppression potential. For example, genomic sequencing of the biocontrol strain *Pantoea ananatis* nfd35 revealed nine secondary metabolite gene clusters and confirmed the role of its siderophores in controlling bitter gourd anthracnose [31]. Notably, a recent study demonstrated that *P. ananatis* BCA19, which strongly antagonizes the pear fire blight pathogen, can directly inhibit pathogen growth through indole production, with its genome enriched in gene clusters encoding diverse antimicrobial compounds [32]. These findings provide a mechanistic explanation for the enrichment of *P. ananatis* observed in this study: BY6 likely induces systemic signaling in Pdpap, reshaping its microbiome and specifically recruiting or promoting colonization and proliferation of beneficial bacteria with both direct antagonistic activity and systemic induction potential in the rhizosphere and phyllosphere.

In summary, this study’s results indicate that BY6 functions as a biocontrol agent through a multilayered, synergistic mechanism that integrates microbiome modulation, physiological and biochemical responses, and gene expression regulation. These coordinated responses collectively enhanced Pdpap’s resistance to pathogen invasion. Future research could focus on elucidating the specific mechanisms underlying endophyte-mediated signaling and community interactions between aboveground (phyllosphere) and belowground (rhizosphere) systems. Addressing these questions will provide a more robust theoretical foundation for developing microbiome-based, sustainable forestry practices.

## 5. Conclusions

This study demonstrates that the endophytic bacterium *B. velezensis* BY6 acts as an effective and multifaceted biocontrol agent against Armillaria root rot in poplar. It reshapes the plant-associated microbiome by enriching beneficial bacterial taxa (e.g., *Pantoea ananatis* and *Actinobacteria*) in both the rhizosphere and phyllosphere. Furthermore, BY6 systemically primes host defense by activating key enzymes (PAL, POD) and reprogramming defense-related signaling pathways, as evidenced by the upregulation of SA/JA marker genes *(PR1*, *JAZ*, *COI1*) and the downregulation of the auxin transporter gene *AUX1*. These coordinated microbial, physiological, and molecular responses collectively enhance poplar resistance, leading to a significant reduction in disease severity. Overall, our findings highlight the potential of harnessing beneficial microbes such as BY6 to develop sustainable, integrated strategies for managing soil-borne diseases in forest ecosystems.

## Data Availability

The original contributions presented in this study are included in the article. Further inquiries can be directed to the corresponding author.
